# Effects of dietary interventions on cardiovascular outcomes: a network meta-analysis

**DOI:** 10.1093/nutrit/nuad080

**Published:** 2023-07-11

**Authors:** Ioannis Doundoulakis, Ioannis T Farmakis, Xenophon Theodoridis, Antonis Konstantelos, Maria Christoglou, Evangelia Kotzakioulafi, Lydia Chrysoula, Antonis Siargkas, Apostolos Karligkiotis, Georgia Kyprianou, Eleni Mastromanoli, Stergios Soulaidopoulos, Stefanos Zafeiropoulos, Christina Antza, Dimitris Tsiachris, Michail Chourdakis

**Affiliations:** First Department of Cardiology, National and Kapodistrian University, “Hippokration” Hospital, Athens, Greece; Center for Thrombosis and Hemostasis, University Medical Center Mainz, Mainz, Germany; Laboratory of Hygiene, Social & Preventive Medicine and Medical Statistics, School of Medicine, Faculty of Health Sciences, Aristotle University of Thessaloniki, Thessaloniki, Greece; Laboratory of Hygiene, Social & Preventive Medicine and Medical Statistics, School of Medicine, Faculty of Health Sciences, Aristotle University of Thessaloniki, Thessaloniki, Greece; Laboratory of Hygiene, Social & Preventive Medicine and Medical Statistics, School of Medicine, Faculty of Health Sciences, Aristotle University of Thessaloniki, Thessaloniki, Greece; Laboratory of Hygiene, Social & Preventive Medicine and Medical Statistics, School of Medicine, Faculty of Health Sciences, Aristotle University of Thessaloniki, Thessaloniki, Greece; Laboratory of Hygiene, Social & Preventive Medicine and Medical Statistics, School of Medicine, Faculty of Health Sciences, Aristotle University of Thessaloniki, Thessaloniki, Greece; Laboratory of Hygiene, Social & Preventive Medicine and Medical Statistics, School of Medicine, Faculty of Health Sciences, Aristotle University of Thessaloniki, Thessaloniki, Greece; Laboratory of Hygiene, Social & Preventive Medicine and Medical Statistics, School of Medicine, Faculty of Health Sciences, Aristotle University of Thessaloniki, Thessaloniki, Greece; Laboratory of Hygiene, Social & Preventive Medicine and Medical Statistics, School of Medicine, Faculty of Health Sciences, Aristotle University of Thessaloniki, Thessaloniki, Greece; Laboratory of Hygiene, Social & Preventive Medicine and Medical Statistics, School of Medicine, Faculty of Health Sciences, Aristotle University of Thessaloniki, Thessaloniki, Greece; First Department of Cardiology, National and Kapodistrian University, “Hippokration” Hospital, Athens, Greece; Elmezzi Graduate School of Molecular Medicine, Northwell Health, Manhasset, New York, USA; Feinstein Institutes for Medical Research at Northwell Health, Manhasset, New York, USA; 3rd Department of Internal Medicine, G.N Papageorgiou, School of Medicine, Aristotle University of Thessaloniki, Thessaloniki, Greece; First Department of Cardiology, National and Kapodistrian University, “Hippokration” Hospital, Athens, Greece; Laboratory of Hygiene, Social & Preventive Medicine and Medical Statistics, School of Medicine, Faculty of Health Sciences, Aristotle University of Thessaloniki, Thessaloniki, Greece

**Keywords:** cardiovascular outcomes, dietary interventions, Mediterranean diet, mortality, network meta-analysis

## Abstract

**Context:**

Next to a large body of epidemiological observational studies showing that the Mediterranean diet (MD) is an important lifestyle determinant of cardiovascular risk, there is less relevant evidence from well-conducted randomized controlled trials (RCTs) with hard cardiovascular outcomes.

**Objective:**

The objective of the study was to identify the most effective dietary intervention for reducing cardiovascular morbidity and mortality.

**Data Sources:**

A systematic approach following PRISMA network meta-analyses reporting guidelines was applied to a search of electronic databases (MEDLINE, Cochrane Central Register of Controlled Trials, and Embase) without language restrictions, supplemented by scanning through bibliographies of studies and meetings’ abstract material. Inclusion criteria were RCTs conducted in an adult population, investigating the effects of different type of diets or dietary patterns on all-cause mortality and cardiovascular outcomes of interest.

**Data Extraction:**

Data extraction for each study was conducted by 2 independent reviewers.

**Data Analysis:**

A frequentist network meta-analysis using a random-effects model was conducted. Death from any cardiovascular cause was defined as the primary outcome. A total of 17 trials incorporating 83 280 participants were included in the systematic review. Twelve articles (n = 80 550 participants) contributed to the network meta-analysis for the primary outcome. When compared with the control diet, only the MD showed a reduction in cardiovascular deaths (risk ratio = 0.59; 95% confidence interval, 0.42–0.82). Additionally, MD was the sole dietary strategy that decreased the risk of major cardiovascular events, myocardial infarction, angina, and all-cause mortality.

**Conclusions:**

MD may play a protective role against cardiovascular disease and death for primary and also secondary prevention.

**Systematic Review Registration:**

Center for Open Science, https://doi.org/10.17605/OSF.IO/5KX83

## INTRODUCTION

Globally, cardiovascular disease (CVD) continues to be the leading cause of mortality and morbidity. High blood pressure measurements, triglyceride and total cholesterol concentrations, and body mass index are among the predisposing factors for CVD.^1^ Dietary factors seem to have the cornerstone role for the prevention of CVD outcomes across Europe.^2^

The Mediterranean diet (MD) is 1 of the most well-studied dietary patterns. Epidemiological evidence since the 1960s has shown that residents of Mediterranean countries had lower rates of cardiovascular mortality in contrast to the northern European populations or the US population.^3–5^ Next to a large body of epidemiological observational studies showing that the MD is an important lifestyle determinant of CVD risk,^3^^,^^6^^,^^7^ there is less relevant evidence from well-conducted randomized controlled trials (RCTs) with hard cardiovascular outcomes. The Prevention of Cardiovascular Disease with a Mediterranean Diet (PREDIMED) study, a large RCT focused on the primary prevention of CVD among patients with a high cardiovascular risk, demonstrated that MD was associated with a lower incidence of major cardiovascular events.^8^

However, a recent umbrella review showed that none of the included dietary interventions could reduce cardiovascular events and only the use of omega-3 fatty acids and folate supplementation might reduce some cardiovascular end points.^9^ Recommendations do not comprehensively address this issue, because they are mostly based on scarce data about dietary interventions with head-to-head comparisons with regard to different dietary interventions.^10–12^ Therefore, in this network meta-analysis (NMA) of RCTs, we synthesized direct and indirect evidence across the literature to identify the most effective dietary intervention for CVD prevention.

## ΜATERIALS AND ΜETHODS

The Preferred Reporting Items for Systematic Reviews and meta-analyses extension statement for network meta-analyses (PRISMA-NMA) were followed in this systematic review and NMA.^13^ A pre-specified protocol has been registered in the Open Science Framework (OSF) database (https://doi.org/10.17605/OSF.IO/5KX83).

### Data sources and searches

The MEDLINE (via PubMed), Cochrane Central Register of Controlled Trials, and Embase databases were used for all searches from inception to May 31, 2022, with no language restrictions. A search strategy was created for PubMed and was accordingly modified for the other electronic databases (see Supplementary Material 1 in the Supporting Information online). Conference abstracts from major nutrition and metabolism conferences and references of relevant studies were screened. The PROSPERO database was searched to retrieve possible similar, ongoing systematic reviews to avoid duplication with this study.^14^^,^^15^

### Inclusion and exclusion criteria

RCTs were included that compared either 2 specific dietary interventions or a dietary intervention with a control diet, regardless of the study design (parallel or crossover) and that reported at least 1 event of the following: all-cause mortality, cardiovascular-related death, myocardial infarction, stroke, angina, heart failure, atrial fibrillation (PICOS criteria are reported at Table 1). Cardiovascular death was set as the primary endpoint. The initial search included a plethora of different dietary interventions, but few of these were used among eligible studies: Mediterranean diet,^8^^,^^16–18^ low-fat diet,^19^^,^^20^ low-protein diet,^21^ low-salt diet,^22^^,^^23^ normal or usual diet.^24^

**Table 1:** Characteristics (PICOS [population, intervention, comparison, outcomes, study design] criteria) of randomized clinical trials^a^ included in this systematic review, in chronological order

Reference	Country	Population	No. of patients	Treatment arms	Primary end point	Follow-up duration
Morrison, 1951^46^	United States	Recent coronary thrombosis and MI	100	Low-cholesterol, low-fat diet Usual diet	All-cause mortality	3 y
Ball, 1965^34^	England	Coronary heart disease	264	Low-fat diet Normal/standard diet	Recurrent MI or CV death	3 y
Woodhill, 1978^42^	Australia	Men; coronary heart disease	458	Usual diet Low-saturated fat, low-cholesterol diet	Death	2–7 y
Singh, 1992^43^	India	Recent acute MI	406	Cardioprotective diet with increased fruit, vegetables, pulses, nuts, and fish consumption and motivational follow-up (MD) Low-fat diet	CV and all-cause mortality	1 y
Renaud, 1995^38^ Lorgeril, 1994^44^	France	Patients recovering from MI	584^38^ 605^44^	Mediterranean α-linolenic acid-rich diet Usual diet	CV mortality and nonfatal MI	27 mo
Lorgeril, 1999^39^	France	Survived a first MI	423	MD Western diet	Nonfatal MI, cardiac death, and their composite	46 mo
Singh, 2002^45^	India	Angina pectoris, MI, or risk factors for CAD	1000	Indo-Mediterranean diet National Cholesterol Education Program step I diet	Fatal or nonfatal MI, sudden cardiac death, and their composite	2 y
Facchini, 2003^36^	United States	Type 2 diabetes	191	Low-iron-available, polyphenol-enriched, carbohydrate-restricted diet Conventional diet	Doubling of serum creatinine level, ESRD, all-cause mortality	3.9 y
Holme, 2006^47^	Norway	High cardiovascular risk, but with no cardiovascular disease	1232	Individualized dietary advice designed to reduce serum total cholesterol level and cigarette consumption (low-fat diet) Usual care	Nonfatal and fatal MI	5 y
Levey, 2006^37^	United States	Nondiabetic kidney disease and moderate decrease of GFR	585	Usual-protein diet Low-protein diet	Decreased GFR	Trial follow-up: 2.2 y Long-term follow-up: 7.3 y
Giannuzzi, 2008^40^	Italy	Recent MI	3241	Multifactorial, continued educational and behavioral program Usual care	Composite end point including CV death, nonfatal MI, nonfatal stroke, hospitalization for angina pectoris or heart failure, and urgent unplanned revascularization procedure	3 y
Tuttle et al, 2008^35^	United States	First MI	101	Low-fat diet MD	Composite end point including all-cause and cardiac mortality, MI, hospital admissions for heart failure, unstable angina, or stroke	46 mo
Wing, 2013^41^	United States	Overweight or obese with type 2 diabetes	5145	An intensive lifestyle intervention that promoted weight loss through decreased caloric intake and increased physical activity Usual care	Composite of death from cardiovascular causes, nonfatal MI, nonfatal stroke, or hospitalization for angina	9.6 y
Estruch, 2018^8^ Martínez-González, 2014 Ruiz-Canela, 2014^32^ Papadaki, 2017^33^	Spain	High cardiovascular risk, but with no cardiovascular disease	7447^8^ 6705 7435^32^ 7403^33^	MD supplemented with extra-virgin olive oil MD supplemented with nuts Reduced-fat diet	MACE New-onset atrial fibrillation Peripheral artery disease events New-onset heart failure	4.8 y
Hua, 2017^23^	China	Hypertensive	12 245	Lifestyle intervention with regular exercise and reduced salt intake Usual care	Composite end point including nonfatal stroke, CV death, and nonfatal MI	3.5 y
Prentice, 2017^20^	United States	Postmenopausal women	48 835	Low-fat eating pattern Usual diet	Breast and colorectal cancer	8.3 y
Delgado-Lista, 2022^48^	Spain	Patients with coronary heart disease	1002	MD Low-fat diet with complex carbohydrates	MACE, revascularization, documented peripheral artery disease	7 y

a All studies are randomized controlled trials according to inclusion criteria.

*Abbreviations:* CAD, coronary artery disease; CV, cardiovascular; ESRD, end-stage renal disease; GFR, glomerular filtration rate; MACE, myocardial infarction, stroke, or cardiovascular mortality; MI, myocardial infarction.

Nonrandomized controlled trials, observational studies, and studies performed with children and adolescents were excluded from this review.

### Study selection

Retrieved articles were imported into a reference manager (EndNote X7). After duplicate removal, 4 pairs of 2 independent reviewers (A. Konstantelos, A. Karligkiotis, A.S., E.K., E.M., G.K., L.C., and M.C.) first assessed titles and abstracts and then evaluated full texts for eligible studies. Any disagreements were adjudicated by an additional reviewer (I.D.).

### Data extraction

Data extraction for each study was conducted by 2 pairs of 2 independent reviewers (I.D., I.F., S.Z., and S.S.), and discrepancies were settled through consensus. A predesigned data collection form was used to extract data on study participants' baseline characteristics, as well as their outcomes. Crossover data were extracted solely from the first period.^25^

### Quality assessment

The revised Cochrane risk-of-bias tool (RoB 2.0) was used by 2 reviewers (S.S. and S.Z.) to independently assess the quality of the included studies.^26^ Any discrepancies were resolved by a third reviewer (I.D.).

### Certainty of the evidence

The Confidence in Network Meta-Analysis approach was applied to assess the certainty of the findings.^27^

### Data synthesis and analysis

To combine direct and indirect evidence across trials, a frequentist NMA with a random-effects model was used. Risk ratios (RRs) with the appropriate 95% confidence intervals (CIs) were used to summarize the evidence. Global approaches, such as a design-by-treatment interaction model,^28^ and local ones, such as analyzing consistency across direct and indirect comparisons with the node-splitting method, were used to measure consistency. The *P* value was used to categorize dietary interventions in a hierarchical order.^29^ Publication bias was evaluated using the comparison-adjusted funnel plot.^30^ The analyses were conducted using the *netmeta* package in R, version 3.6.3 (the R Project for Statistical Computing).

## RESULTS

### Overview of studies

**Figure 1:**
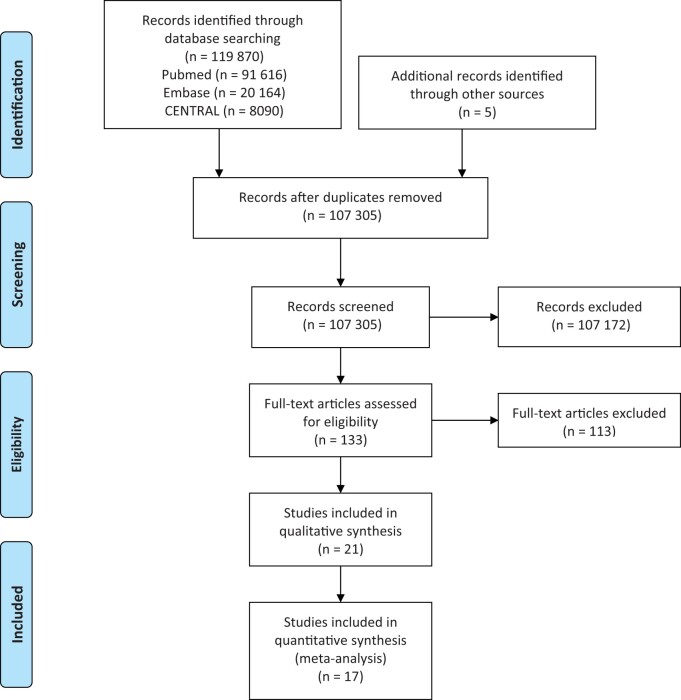
PRISMA flowchart of the study selection process.

After the removal of duplicates, 107 305 records were evaluated on the basis of their title and abstract (Figure 1). Eventually, 17 studies of a total of 83 280 participants met the inclusion criteria for this systematic review.^8^^,^^20^^,^^23^^,^^31–48^ The summaries of the included studies are listed in Table 1. The risk of bias was judged as low in 6 studies, with some concerns in 4 studies, and was judged high in 7 studies (see Supplementary Figure S1 in the Supporting Information online). Supplementary Table S1 in the Supporting Information online provides the dietary details in each interventional arm.

### Network meta-analysis

#### Cardiovascular death

**Figure 2:**
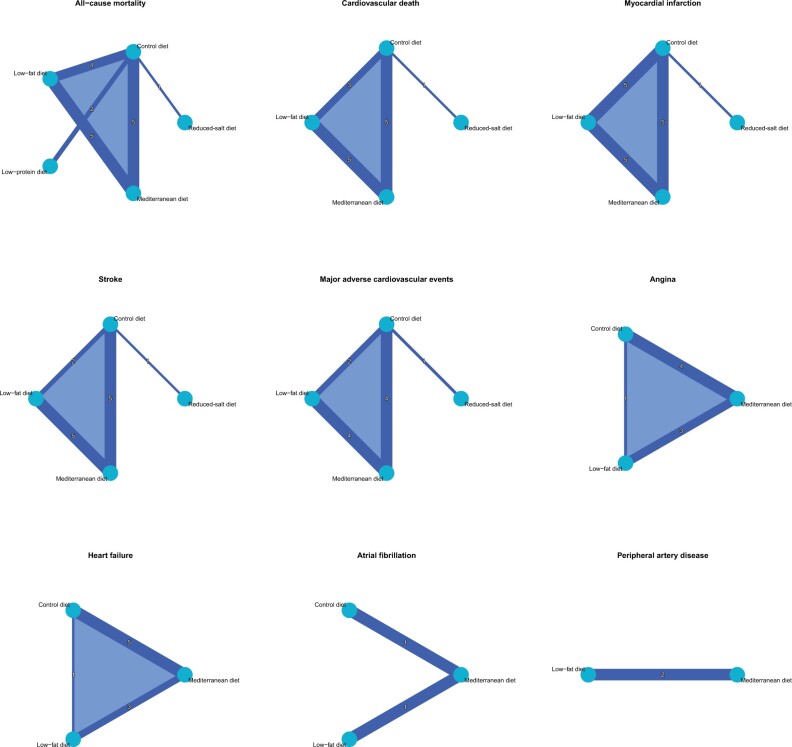
The network graphs of interventions for the outcomes.

**Figure 3:**
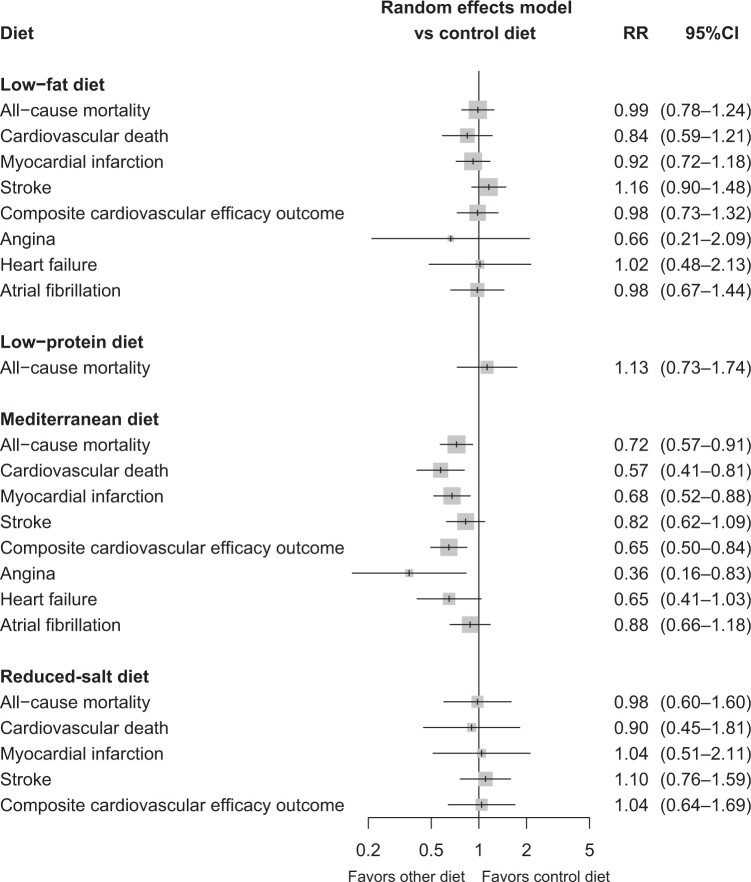
Forest plot of the network estimates of the potent dietary intervention against control diet for the outcomes.

A total of 12 studies (n = 80 550 patients) contributed to the NMA.^8^^,^^20^^,^^23^^,^^35^^,^^39–41^^,^^43–46^^,^^48^Figure 2 visualizes the NMA graph. Only the MD decreased the risk of cardiovascular death when compared with the control diet (RR = 0.59; 95%CI, 0.42–0.82) (Figure 3). There was a difference between the low-fat diet and MD (RR = 1.46; 95%CI, 1.07–1.99), but not with MD and a reduced-salt diet (RR = 0.65; 95%CI, 0.31–1.38) (Table 2). MD ranked best (*P* = 0.9532), followed by the low-fat diet (*P* = 0.4565), reduced-salt diet (*P* = 0.3997), and the control diet (*P* = 0.1905). No disagreement was detected between direct and indirect evidence with the node-splitting method. Evidence of publication bias was absent in the network (Egger’s test, *P* = 0.7812). Supplementary Material 2 in the Supporting Information online provides further information on consistency, the funnel plot, and the influence of individual studies on the network of the primary outcome.

**Table 2:** League table showing the results of the network meta-analyses comparing the effects of interventions for the primary outcome^a^

Control diet	1.23 (0.79–1.92)	1.64 (1.10–2.45)	1.11 (0.57–2.18)
1.17 (0.83–1.65)	Low-fat diet	1.51 (1.05–2.16)	
**1.70 (1.23–2.37)**	**1.46 (1.07–1.99)**	Mediterranean diet	
1.11 (0.57–2.18)	0.95 (0.45–2.03)	0.65 (0.31–1.38)	Reduced-salt diet

a The upper triangle contains the pooled effect sizes expressed as risk ratios and their 95% confidence intervals of the direct comparisons available in our network. The lower triangle of the matrix contains the estimated effect sizes expressed as risk ratios and their 95% confidence intervals for each comparison. Significant results are in bold.

In the subgroup analysis of studies including exclusively patients with established CVD (n = 7 studies; n = 5878 patients),^35^^,^^39^^,^^40^^,^^43^^,^^44^^,^^46^^,^^48^ MD resulted in a reduction in cardiovascular death versus the control diet (RR = 0.39; 95%CI, 0.25–0.63), whereas a low-fat diet did not (RR = 0.62; 95%CI, 0.35–1.08).

#### Secondary outcomes

Fifteen studies (n = 81 948 patients) contributed to the network for the all-cause mortality outcome (see Supplementary Material 3 in the Supporting Information online).^8^^,^^20^^,^^23^^,^^34–37^^,^^39–45^^,^^48^ Compared with the control diet, only the MD reduced the mortality risk (RR = 0.73; 95%CI, 0.58–0.92) (Figure 3). There was a difference between the low-fat diet and the MD (RR = 1.36; 95%CI, 1.10–1.68), but not between the MD and reduced-salt (RR = 0.74; 95%CI, 0.44–1.25), nor between the MD and the low-protein diet (RR = 1.53; 95%CI, 0.95–2.47) (see Table S5A in the Supporting Information online). MD ranked best (*P* = 0.9554) in terms of effect on secondary outcomes, followed by the reduced-salt diet (*P* = 0.4586), low-fat diet (*P* = 0.4262), the control diet (*P* = 0.4140), and the low-protein diet (*P* = 0.2459). No disagreement was detected between direct and indirect evidence with the node-splitting method. Evidence of publication bias was absent in the network (Egger’s test, *P* = 0.3166). In the subgroup analysis of studies including exclusively patients with established CVD (n = 8 studies; n = 6500 patients),^34^^,^^35^^,^^39^^,^^40^^,^^42–44^^,^^48^ MD resulted in reduced mortality rates in comparison with a control diet (RR = 0.61; 95%CI, 0.43–0.87) and low-fat diet (RR = 0.62; 95%CI, 0.44–0.87).

Fourteen studies (n = 82 046 patients) contributed to the network for the myocardial infarction outcome (see Supplementary Material 4 in the Supporting Information online).^8^^,^^20^^,^^23^^,^^34^^,^^35^^,^^39–41^^,^^43–48^ Compared with the control diet, only the MD reduced the mortality risk (RR = 0.68; 95%CI, 0.52–0.88) (Figure 3). There was a difference between low-fat diet and the MD (RR = 1.36; 95%CI, 1.05–1.75), but not between the MD and the reduced-salt diet (RR = 0.65; 95%CI, 0.31–1.38) (see Table S6A in the Supporting Information online). MD ranked first (*P* = 0.9529) for the effect on myocardial infarction outcome, followed by the low-fat diet (*P* = 0.4653), reduced-salt diet (*P* = 0.3188), and the control diet (*P* = 0.2630). With reference to the subgroup analysis of studies that included exclusively patients with established CVD (n = 8 studies; n = 6500 patients),^34^^,^^35^^,^^39^^,^^40^^,^^42–44^^,^^48^ no difference was observed between the dietary interventions.

Eleven studies (n = 80 429 patients) contributed to the NMA on stroke outcome (see Supplementary Material 5 in the Supporting Information online).^8^^,^^20^^,^^23^^,^^35^^,^^38–41^^,^^43^^,^^45^^,^^48^ Compared with the control diet, no difference was observed between the dietary interventions (Figure 3). A low-fat diet was associated with an increased risk for stroke, compared with the MD (RR = 1.34; 95%CI, 1.05–1.70) (see Table S5 in the Supporting Information online). In the subgroup analysis of studies including exclusively patients with established CVD (n = 6 studies; n = 5757 patients),^35^^,^^38–40^^,^^43^^,^^48^ the same results were observed. According to Egger’s test, there was publication bias (*P* = 0.0118) regarding to this outcome.

Nine studies (n = 78 843 patients) contributed to the network for the major cardiovascular event outcome (myocardial infarction, stroke, or mortality from cardiovascular causes; see Supplementary Material 6 in the Supporting Information online).^8^^,^^20^^,^^23^^,^^35^^,^^39–41^^,^^43^^,^^45^ Compared with the control diet, only the MD reduced the mortality risk (RR = 0.64; 95%CI, 0.49–0.84) (Figure 3). Furthermore, a low-fat diet was associated with an increased risk for stroke compared with the MD (RR = 1.53; 95%CI, 1.18–1.97) (see Table S6 in the Supporting Information online). MD ranked as the best intervention in terms of effect on major cardiovascular events (*P* = 0.9851), whereas the reduced-salt diet was the worst dietary plan (*P* = 0.2948). There was a difference between the MD and low-fat diet (RR = 1.53; 95%CI, 1.18–1.97), but  not between the MD and the reduced-salt diet (RR = 0.61; 95%CI, 0.35–1.07).

Six studies (n = 7659 patients) contributed to the NMA for the angina outcome (see Supplementary Material 7 in the Supporting Information online).^35^^,^^38^^,^^39^^,^^41^^,^^43^^,^^45^ Compared with the control diet, only the MD reduced the mortality risk (RR = 0.36; 95%CI, 0.16–0.84) (Figure 3). There was no difference between the low-fat diet and the MD (RR = 1.83; 95%CI, 0.72–4.65) (see Table S7 in the Supporting Information online).

Seven studies contributed to the NMA for the heart failure outcome (see Supplementary Material 8 in the Supporting Information online),^33^^,^^35^^,^^38–41^^,^^45^ and 2 studies contributed to the NMA for the atrial fibrillation outcome (see Supplementary Material 9 in the Supporting Information online).^31^^,^^40^ There was no differences among the MD, low-fat diet, and control diet (see Table S8 in the Supporting Information online).

### Grading of evidence

Moderate confidence evidence demonstrated that only the MD managed to decrease the cardiovascular mortality risk (see Supplementary Table S9 in the Supporting Information online) when compared with control diets.

## DISCUSSION

In this study, the comparative efficacy of dietary interventions for CVD prevention was assessed by an NMA. Moderate confidence evidence suggests that, compared with the control diet, only the MD decreased the cardiovascular mortality risk. In addition, the MD was the only effective intervention for decreasing all-cause mortality, myocardial infarction, angina, and major cardiovascular events.

The MD contains higher quantities of fruit, vegetables, nuts, fish, and olive oil.^17^ These quantities have beneficial effects on cardiometabolic markers, endothelial function, and inflammation, decreasing the incidence of cardiovascular outcomes.^49^ A recent study showed that greater amounts of olive oil consumption reduce total CVD and deaths from cancer.^50^ Several studies over the past decades found that the MD was correlated with favorable outcomes, such as a decreased atherosclerosis progression and improved quality of life.^51–53^ Furthermore, the latest European guidelines recommend an MD or similar diet to lower the risk of CVD.^12^ Nonetheless, it should be highlighted that this evidence is not supported by well-conducted, head-to-head comparisons (with few exceptions)^8^^,^^35^^,^^37^^,^^48^ among dietary interventions (trials or NMAs).

According to the NMA findings, the MD is the only effective intervention for the primary and secondary prevention of CVD. Results of a previous RCT with 423 patients with myocardial infarction indicated that the MD had a positive effect on myocardial infarction 4 years after its first incidence.^39^ Moreover, Estruch et al^8^ provided data on patients with a poor cardiac profile. The repercussion of significant cardiovascular outcomes in this study was lower among those assigned to the intervention arms in comparison with those assigned to a reduced-fat diet. Indeed, evidence from cohort studies has shown that greater amounts of olive oil intake were also correlated with beneficial outcomes.^50^ The retracted and republished PREDIMED trial results showed that there was no alteration in the studied outcomes, even after the reanalysis of the data. It was concluded that participants assigned to the MD arms had almost 30% lower incidence of CVD in contrast to individuals who received a control diet. Indeed, this NMA confirmed these results compared with low-fat, reduced-salt, and usual diets.

None of the included RCTs in this review evaluated the cardiovascular benefits of plant-based diets. This intervention might have a key role in CVD prevention. In previous cohorts, lower levels of blood pressure and low-density lipoprotein cholesterol, and reduced incidence of type 2 diabetes mellitus were associated with this diet.^54^^,^^55^ A recent meta-analysis of observational studies showed that plant-based diets were associated with a lower risk of CVD.^56^ Thus, European guidelines have included this intervention as a food pattern for people at high cardiovascular risk.^12^

Although this NMA provides evidence in the field, the findings must be interpreted with caution. Among the included studies, some were published before the introduction of the Consolidated Standards of Reporting Trials statement; hence, they may lack rigor of methodology. Although the MD remains the best option in the subgroup analysis of studies including exclusively patients with established CVD, it is unknown whether the benefits of diets are evident in other specific populations, such as elderly patients or patients with diabetes mellitus. An additional limitation identified in the studies included in our NMA was that participants following the MD diet achieved greater weight loss than did population groups assigned to other dietary interventions. Hence, the overall reduction of CVD risk may be attributed to changes in body weight and not to dietary interventions per se. Some of the included trials were published before 2000 and had methodological issues.

## CONCLUSION

The MD may have a protective role against cardiovascular death and for primary and secondary prevention of CVD.

## Supplementary Material

nuad080_Supplementary_Data

## Data Availability

The data underlying this article are available in the article and its online supplementary material, and the review protocol can be found in https://doi.org/10.17605/OSF.IO/5KX83.

## Supporting Information

The following Supporting Information is available through the online version of this article at the publisher’s website.

*Supplementary Material 1* **Search strategy**

*Supplementary Figure S1* **Quality assessment of included studies**

*Supplementary Table S1* **Dietary details in each interventional arm**

*Supplementary Material 2* **Assessment of inconsistency and funnel plot for the primary outcome**

*Supplementary Material 3* **Network meta-analysis of mortality interventions**

*Supplementary Material 4* **Network meta-analysis of interventions for myocardial infarction**

*Supplementary Material 5* **Network meta-analysis of interventions for stroke**

*Supplementary Material 6* **Network meta-analysis of interventions for major cardiovascular events (myocardial infarction, stroke, or death from cardiovascular causes) outcome**

*Supplementary Material 7* **Network meta-analysis of interventions for the angina outcome**

*Supplementary Material 8* **Network meta-analysis of interventions for the heart failure outcome**

*Supplementary Material 9* **Network meta-analysis of interventions for the atrial fibrillation outcome**

*Supplementary Table S9* **Grading of evidence for the primary outcome**
