# Supplementary material for: Effects of dietary interventions on cardiovascular outcomes: a network meta-analysis
Source: Nutr Rev. 2023 Jul 11;82(6):715–25. doi: 10.1093/nutrit/nuad080 (PMC11082588; doi:10.1093/nutrit/nuad080)
Supplement: nuad080_Supplementary_Data [file nuad080_supplementary_data.zip › nuad080_Supplementary_Data/Supplementary data.docx]

**Supplementary material 1:** Search strategy

SEARCH SYNTAX and SEARCH STRING (in PubMed) for RCTs

1. mediterranean diet
2. vegetarian diet
3. plant based diet
4. plant-based diet
5. vegan diet
6. lacto vegetarian diet
7. ovo vegetarian
8. ovo-lacto vegetarian diet
9. pescatarian diet
10. fruitarian diet
11. semi vegetarian diet
12. raw food diet
13. intermittent fasting
14. 5:2 diet
15. alternate day fasting
16. time restricted feeding
17. high carbohydrate diet
18. high carb diet
19. low carbohydrate diet
20. low carb diet
21. low fat diet
22. high fat diet
23. high protein diet
24. low protein diet
25. DASH diet
26. dietary approaches to stop hypertension
27. ketogenic diet
28. low calorie diet
29. hypocaloric diet
30. low sodium diet
31. salt restricted diet
32. high fiber diet
33. high fibre diet
34. fat restricted diet
35. calorie restricted diet
36. TLC diet
37. therapeutic lifestyle changes diet
38. AHA diet
39. American heart association diet
40. cholesterol restricted diet
41. diabetic diet
42. low glycemic load diet
43. low glycemic index diet
44. high glycemic load diet
45. high glycemic index diet
46. high residue diet
47. low residue diet
48. FODMAP diet
49. milk egg wheat soya free diet
50. low lactose diet
51. renal diet
52. low phosphate diet
53. eurodiet
54. eskimo diet
55. inuit diet
56. Scarsdale diet
57. McDougall diet
58. Okinawa
59. gluten free diet
60. MIND diet
61. Ramadan diet
62. EAT-Lancet diet
63. Atkins diet
64. cambridge diet
65. dukan diet
66. harvard diet
67. mayo-clinic diet
68. weight watchers
69. ornish diet
70. zone diet
71. paleo diet
72. Paleolithic diet
73. nordic diet
74. WIC
75. cretan diet
76. cuisine of Mediterranean
77. western diet
78. typical American diet
79. standard American diet
80. greek diet
81. asian diet
82. 1 OR 2 OR 3 OR 4 OR 5 OR 6 OR 7 OR 8 OR 9 OR 10 OR 11 OR 12 OR 13 OR 14 OR 15 OR 16 OR 17 OR 18 OR 19 OR 20 OR 21 OR 22 OR 23 OR 24 OR 25 OR 26 OR 27 OR 28 OR 29 OR 30 OR 31 OR 32 OR 33 OR 34 OR 35 OR 36 OR 37 OR 38 OR 39 OR 40 OR 41 OR 42 OR 43 OR 44 OR 45 OR 46 OR 47 OR 48 OR 49 OR 50 OR 51 OR 52 OR 53 OR 54 OR 55 OR 56 OR 57 OR 58 OR 59 OR 60 OR 61 OR 62 OR 63 OR 64 OR 65 OR 66 OR 67 OR 68 OR 69 OR 70 OR 71 OR 72 OR 73 OR 74 OR 75 OR 76 OR 77 OR 78 OR 79 OR 80 OR 81
83. ischemic attack
84. peripheral vascular disease
85. death
86. cardiovascular death
87. cardiovascular mortality
88. all-cause mortality
89. all cause mortality
90. cerebrovascular disease
91. arrythmia
92. cardiovascular disease
93. coronary heart disease
94. coronary artery disease
95. atrial fibrillation
96. heart failure
97. angina
98. heart attack
99. stroke
100. myocardial infarction
101. ischemic heart disease
102. mortality
103. 83 OR 84 OR 85 OR 86 OR 87 OR 88 OR 89 OR 90 OR 91 OR 92 OR 93 OR 94 OR 95 OR 96 OR 97 OR 98 OR 99 OR 100 OR 101 OR 102
104. 82 AND 103

**Supplementary material 2:** Quality assessment of included studies


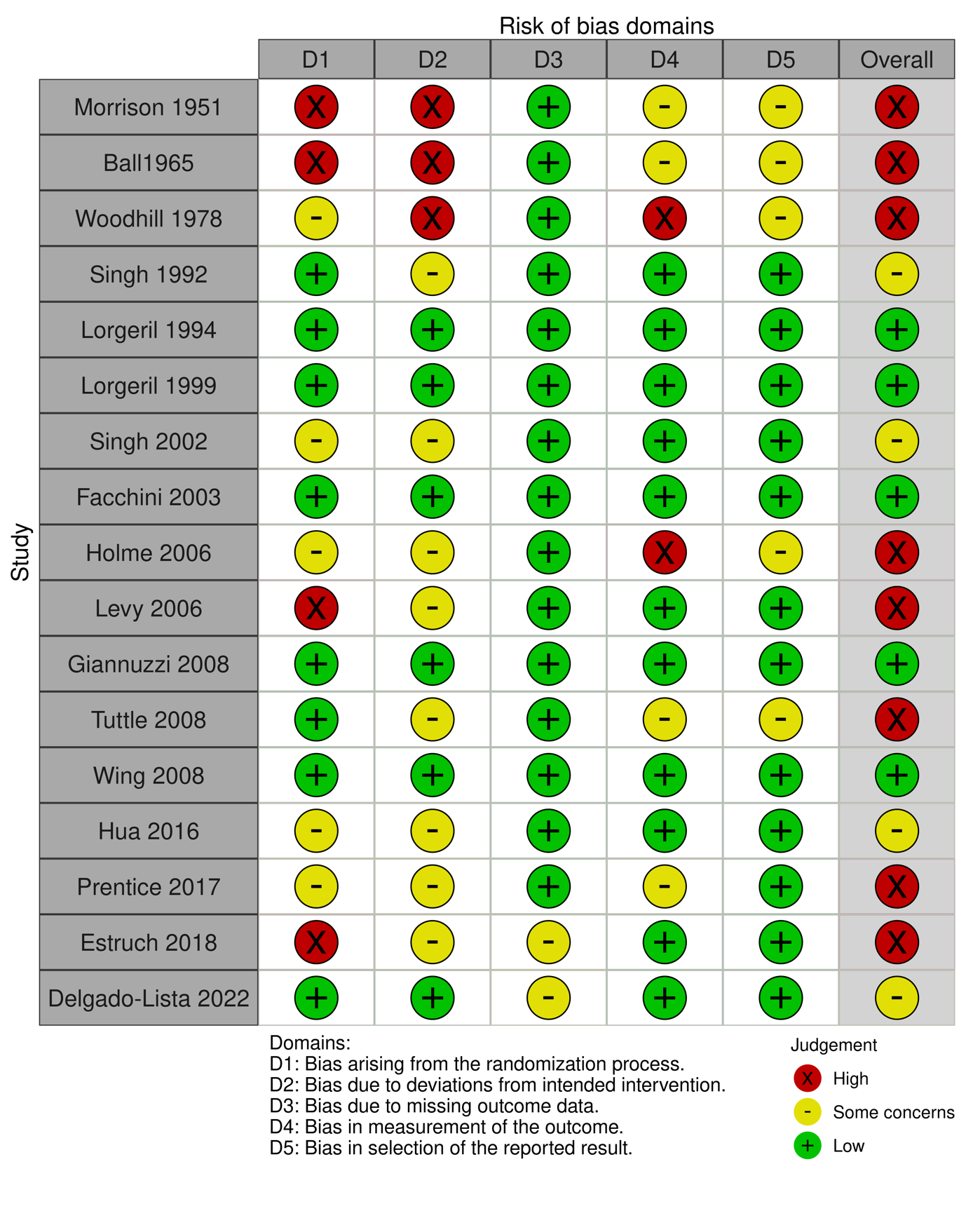


**Supplementary material 3.** Dietary details in each interventional arm.

| **First Author, Year (Country)** | **Dietary Details** | **Carbohydrates** | **Protein** | **Fats** | **Definition of category** |
| --- | --- | --- | --- | --- | --- |
| *Morrison, 1951*  *(USA)* | Low fat about 20 g fat/day | 230 g | 86 g | 23 g | LF |
|  | Average American cholesterol and fat intake |  |  | 80-160 g | Control |
| *Ball, 1965*  *(England)* | The daily allowance included 14 g (1/2 oz.) butter, 84 g (3 oz.) of meat, 1 egg, 56 g (2 oz.) cottage cheese, and skimmed milk. The nature of the fat consumed was not altered, nor were any additional unsaturated fats given  Hypocaloric if overweight (1850-2000 kcal) |  |  | 43-45 | LF |
|  | Usual diet  Hypocaloric if overweight  Reduction in carbohydrates not fats (2300-2600) |  |  | 100-125 | Control |
| *Woodhill, 1978*  *(Australia)* | Usual diet group was not given specific dietary instruction  Restriction in kcal if overweight | 40% | 15% | 38%  SFA 13%  MUFA 14%  PUFA 9% | Control |
|  | Reduced saturated fat intake | 40% | 15% | 38%  SFA 10%  Dietary cholesterol 300 mg or less  PUFA 15%  MUFA 11% | LF |
| *Singh, 1992*  *(India)* | Fat reduced diet. With more fruits grains and vegetables and nuts more intensively advice  Meat, eggs, hydrogenated oils, butter and clarified butter were replaced with vegetarian meat substitutes and soya bean, sunflower and ground nut oils. Target for at least 400g/day fruits and vegetables egg 4-5 /week and meat 1-2/week | 54% | 13% | 24%  SFA 7%  MUFA 8%  PUFA% | MDD |
|  | One time advice for fat reduction-AHA diet.  Fat modified diet (meat, eggs, hydrogenated oils, butter and clarified butter were replaced with vegetarian meat substitutes and soya bean, sunflower and ground nut oils) | 39% | 15% | 28-29%  SFA 11%  MUFA 10%  PUFA 7% | LF |
| *Renaud, 1995/ Lorgeril, 1994*  *(France)* | Adopt a Mediterranean-type diet: more bread, more root vegetables and green vegetables, more fish, less meat (beef, lamb, and pork to be replaced with poultry), no day without fruit, and butter and cream to be replaced with margarine supplied by the study.  Because the patients would not accept olive oil-traditional to the Mediterranean diet-as the only fat, a rapeseed (canola) oil-based margarine with a composition comparable to olive oil with 15 % saturated fatty acids,48% oleic acid but 5-4% 18:1trans. However, it was slightly higher in linoleic (16-4 vs 8-6%) and more so in alpha linolenic acid (4-8 vs 0-6%) |  | 17 g | 28-30%  SFA 7%  Omega-3 0.8% | MD |
|  | Usual diet  No dietary advice |  | 16 g | 32-33%  SFA 12%  Omega-3 0.27% | Control |
| *Lorgeril, 1999*  *(France)* | Follow a Mediterranean type diet  Detailed and individualized dietary instructions for each patient | 18.6 g fiber | 16 g | 30%  SFA 8%  PUFA 4.6%  Omega-3 0.84% | MD |
|  | No dietary advice, recommendation for prudent diet by physician | 15.5 g fiber | 16-17 g | 33-34%  SFA 11-12%  PUFA 6.10%  Omega-3 0.29% | Control |
| *Singh, 2002*  *(India)* | At least 400–500 g of fruits, vegetables, and nuts per day, (ie, 250–300 g of fruit, 125–150 g of vegetables, and 25–50 g of walnuts or almonds). This group was also encouraged to eat 400–500 g of whole grains, legumes, rice, maize, and wheat) daily, as well as mustard seed or soy bean oil, in three to four servings per day two-thirds of patients were vegetarian. The remaining third ate two to five eggs, and one to two portions of meat a week. All participants consumed milk, butter, clarified butter (Indian ghee), and trans fatty acids (vegetable ghee made from partly hydrogenated oils). Vegetarian participants consumed more milk, vegetable ghee, peanut oil, and clarified butter than did non- vegetarians | 57-60% | 14-15% | 26-28%  SFA 8%  MUFA 10%  PUFA 8%  Omega-3 1.79% | MD |
|  | Less than 30% of energy comes from total fat, less than 10% from saturated fat, and that less than 300 mg of cholesterol is consumed per day. two-thirds of patients were vegetarian. The remaining third ate two to five eggs, and one to two portions of meat a week. All participants consumed milk, butter, clarified butter (Indian ghee), and trans fatty acids (vegetable ghee made from partly hydrogenated oils) | 56-57% | 14-15% | 28-29%  SFA 12%  MUFA 8-10%  PUFA 7%  Omega-3 0.53-0.78 % | LF |
| *Facchini, 2003*  *(USA)* | 50% reduction of carbohydrates from the previous intake, substitution of iron-enriched red meats with white meats and with protein -enriched food items known to inhibit iron absorption. Elimination of all beverages except for tea, water, red wine. Milk for breakfast. Use of EVOO | 35% | 25-30% | 30% | Control |
|  | Protein restricted 0.8 g/kg diet isocaloric for IBW, no recommendations for beverages. Limit sucrose beverages | 65% | 10% | 25% | LP |
| *Holme, 2006*  *(Norway)* | Participants with high cholesterol concentrations were asked to reduce consumption of saturated fat and slightly increase polyunsaturated fat. Overweight participants and those with high triglyceride concentrations were asked to reduce body weight and total energy intake (mainly by reducing sugar, sweet drinks, chocolate, alcohol and total fat). For breakfast and evening meals, participants were asked to consume fiber-rich bread with no fat or a thin layer of highly polyunsaturated low-fat margarine. Preferred spreads for bread included fish, fruit, vegetables, low sugar jam, and low-fat meats and cheeses. Skimmed milk and no more than one egg per week were recommended. For main meals, subjects were advised to use lean meat, fish and low-fat fish products together with potatoes and vegetables |  |  |  | LF |
|  | Usual diet |  |  |  | Control |
| *Levey, 2006*  *(USA)* | Usual protein 1.3 g/kg/day |  |  |  | Control |
|  | Low protein 0.58 g/kg/day  65% of animal sources |  |  |  | LP |
| *Giannuzzi, 2008*  *(Italy)* | MD  Adopt a healthy Mediterranean style diet |  |  |  | MD |
|  | Usual care  Letter to family physician about secondary prevention goals |  |  |  | Control |
| *Tuttle, 2008*  *(USA)* | Mediterranean style diet | 50% | 10-20% | 30-40%  MUFA 20-25% Omega-3 >0.75% SFA≤7% Cholesterol ≤200 mg | MD |
|  | AHA Step II Diet | 55-60% | 10-20% | <30%  MUFA 10-15%  Omega-3 0.3-0.45%  SFA ≤7%  Cholesterol ≤200 mg | LF |
| *Wing, 2013*  *(USA)* | 1200-1800 kcal  Use of meal replacements & 175 min of moderate intensity exercise | ~45-55% | >15% | <30% | MD |
|  | Receive diabetes education and support  Diabetes support and education featured three group sessions per year focused on diet, exercise, and social support |  |  |  | Control |
| *Estruch, 2018/ Martinez-Gonzales, 2014/ Ruiz-canela, 2014/ Papadaki, 2017*  *(Spain)* | 50 g or more of extra virgin oil per day (4 tbsp),  b) consumption of ≥2 daily servings of vegetables (at least one of them as fresh vegetables in a salad), without counting garnishing c) ≥2-3 daily serving of fresh fruits (including natural juices); d) ≥3 weekly servings of legumes; e) ≥3 weekly servings of fish or seafood (at least one serving of fatty fish); f) ≥3 weekly servings of nuts or seeds; g) select white meats (poultry without skin or rabbit) instead of red meats or processed meats (burgers, sausages); h) cook regularly (at least twice a week) with tomato, garlic and onion adding or no other aromatic herbs, and dress vegetables, pasta, rice and other dishes with tomato, garlic and onion adding or no other aromatic herbs | NA | NA | Ad libitum  40% (20% MUFA) | MD |
|  | 30 g nuts/day  30 g mixes nuts per day (15 g walnuts, 7.5 g hazelnuts, 7.5 g almonds) | NA | NA | Ad libitum  40% (20% MUFA) | MD |
|  | Negative recommendations are also given to eliminate or drastically limit the consumption of the following foods: cream, butter, margarine, cold meat, paté, duck, carbonated and/ or sugared beverages, pastries, industrial bakery products (such as cakes, donuts or cookies), industrial desserts (puddings, custard), French fries or potato chips, and out-of-home precooked cakes and sweets | NA | NA | <2 tbsp olive oil | Control |
| *Hua, 2016*  *(China)* | Regular exercise and healthy diet  Reduction 5 kg or less  Reduce salt intake  Reduce smoking  Limit alcohol  3 or more 30 min moderate intensity exercise  Increased <6 g/day consumption of fruits and vegetables and reduced salt intake |  |  |  | Reduced salt |
|  | Usual diet |  |  |  | Control |
| *Prentice, 2017*  *(USA)* | Reduction in total fat intake 20%  Increase in vegetable and fruit intake to 5 servings/day and in grain intake to 6 servings/day | 56-59% | 16-18% | 23-27% | LF |
|  | Printed health-related materials only | 47-48% | 16-18% | 34-35% | Control |
| *Delgado-Lista, 2022*  *(Spain)* | A minimum of 35% of the calories as fat (22% monounsaturated fatty acids, 6% polyunsaturated fatty acids, and <10% saturated fat), 15% proteins, and a maximum of 50% carbohydrates | <50% | 15% | 35 % (MUFA 22%, PUFA 6%, SFA<10%) | MD |
|  | High complex carbohydrates diet, comprising less than 30% of total fat (<10% saturated fat, 12–14% monounsaturated fatty acids, and 6–8% polyunsaturated fatty acids), 15% protein, and a minimum of 55% carbohydrates | >55% | 15% | <30% (MUFA 12-14% , PUFA 6-8% SFA<10%) | LF |
| AHA: American Heart Association; Control: Control Diet; EVOO: Extra Virgin Olive Oil; IBW: Ideal Body Weight; LC: Low carbohydrate; LF: Low-fat diet; LP: Low Protein; MD: Mediterranean Diet; MUFA: Mono Unsaturated Fatty Acids; PUFA: Poly Unsaturated Fatty Acids; Reduced salt: Reduced Salt Diet; SFA: Saturated Fatty Acids | | | | | |

**Supplementary material 4:** Assessment of inconsistency and funnel plot for the primary outcome

***Table S4A.*** *Node-splitting method for assessment of inconsistency in network meta-analysis.*

| **Comparison** | **k** | **prop** | **NMA** | **Direct** | **Indirect** | **RoR** | **z** | **p-value** |
| --- | --- | --- | --- | --- | --- | --- | --- | --- |
| Control diet : Low fat diet | 3 | 0.59 | 1.18 | 1.25 | 1.09 | 1.14 | 0.36 | 0.72 |
| Control diet : Mediterranean diet | 5 | 0.68 | 1.75 | 1.69 | 1.88 | 0.90 | -0.29 | 0.77 |
| Control diet : Reduced salt diet | 1 | 1.00 | 1.11 | 1.11 | - | - | - | - |
| Low fat diet : Mediterranean diet | 5 | 0.74 | 1.48 | 1.52 | 1.36 | 1.12 | 0.30 | 0.77 |
| Low fat diet : Reduced salt diet | 0 | 0 | 0.94 | - | 0.94 | - | - | - |
| Mediterranean diet : Reduced salt diet | 0 | 0 | 0.64 | - | 0.64 | - | - | - |
| **k: Number of studies providing direct evidence, prop: Direct evidence proportion, NMA: Estimated treatment effect (HR) in network meta-analysis, Direct: Estimated treatment effect (HR) derived from direct evidence, Indirect: Estimated treatment effect (HR) derived from indirect evidence, RoR: Ratio of ratios (direct versus indirect), z: z-value of test for disagreement (direct versus indirect), p-value: p-value of test for disagreement (direct versus indirect)* | | | | | | | | |

*
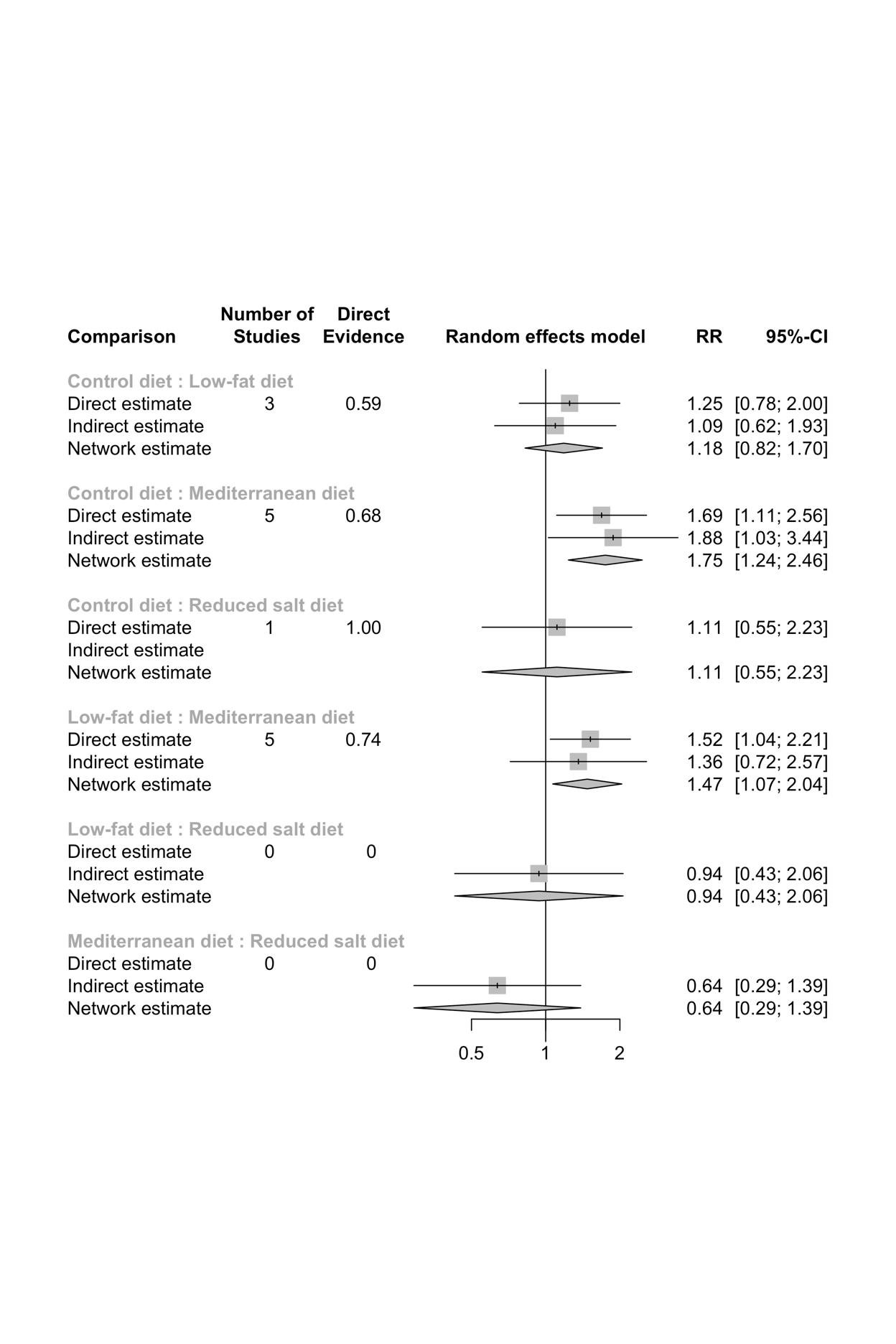
*

***Figure S4A.*** *Forest plot of the direct and indirect evidence for the individual comparisons.*


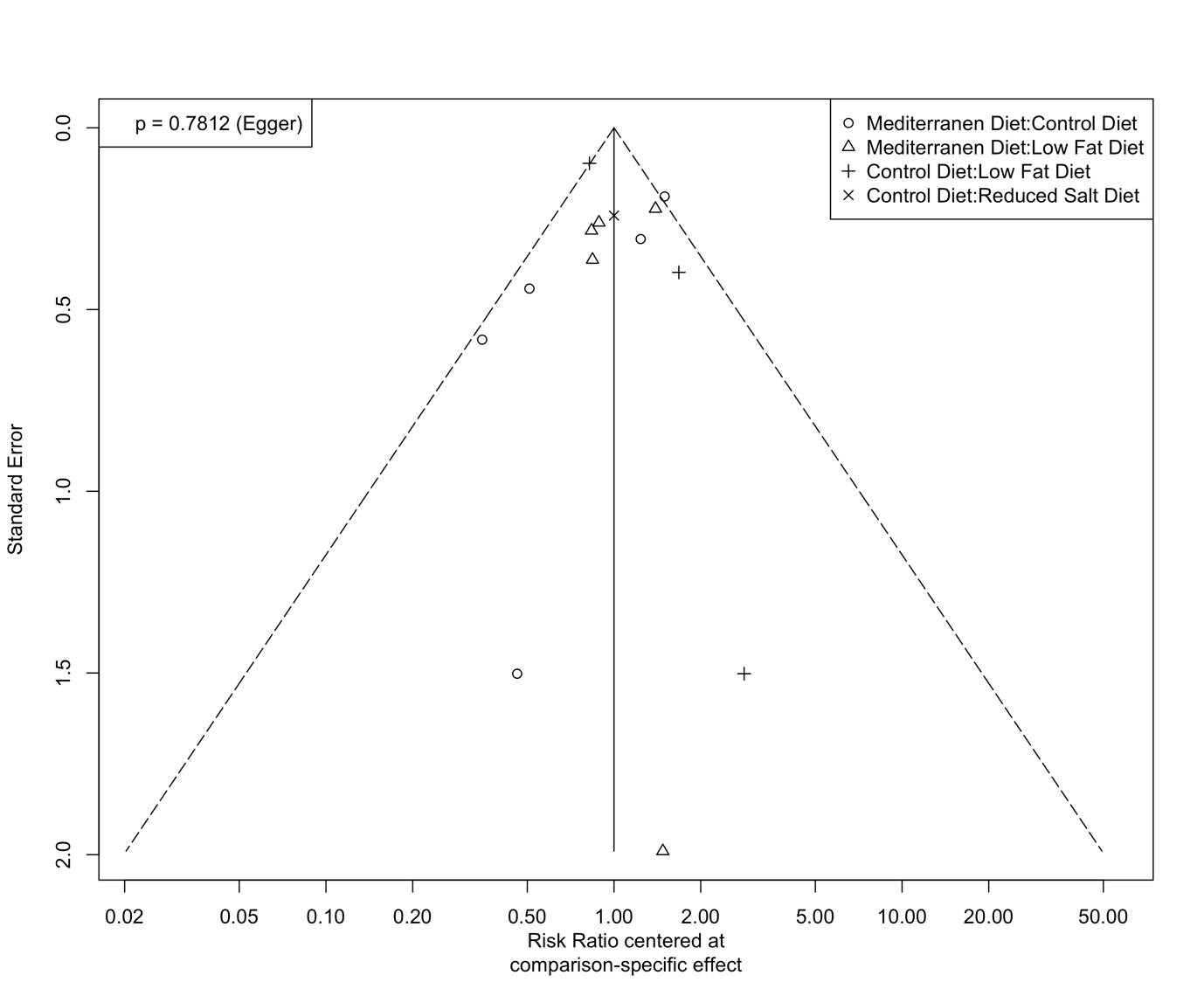


***Figure S4B.*** *Funnel plot of studies contributing to the network for the primary outcome.*

**Supplementary material 5:** Network meta-analysis of interventions for the mortality


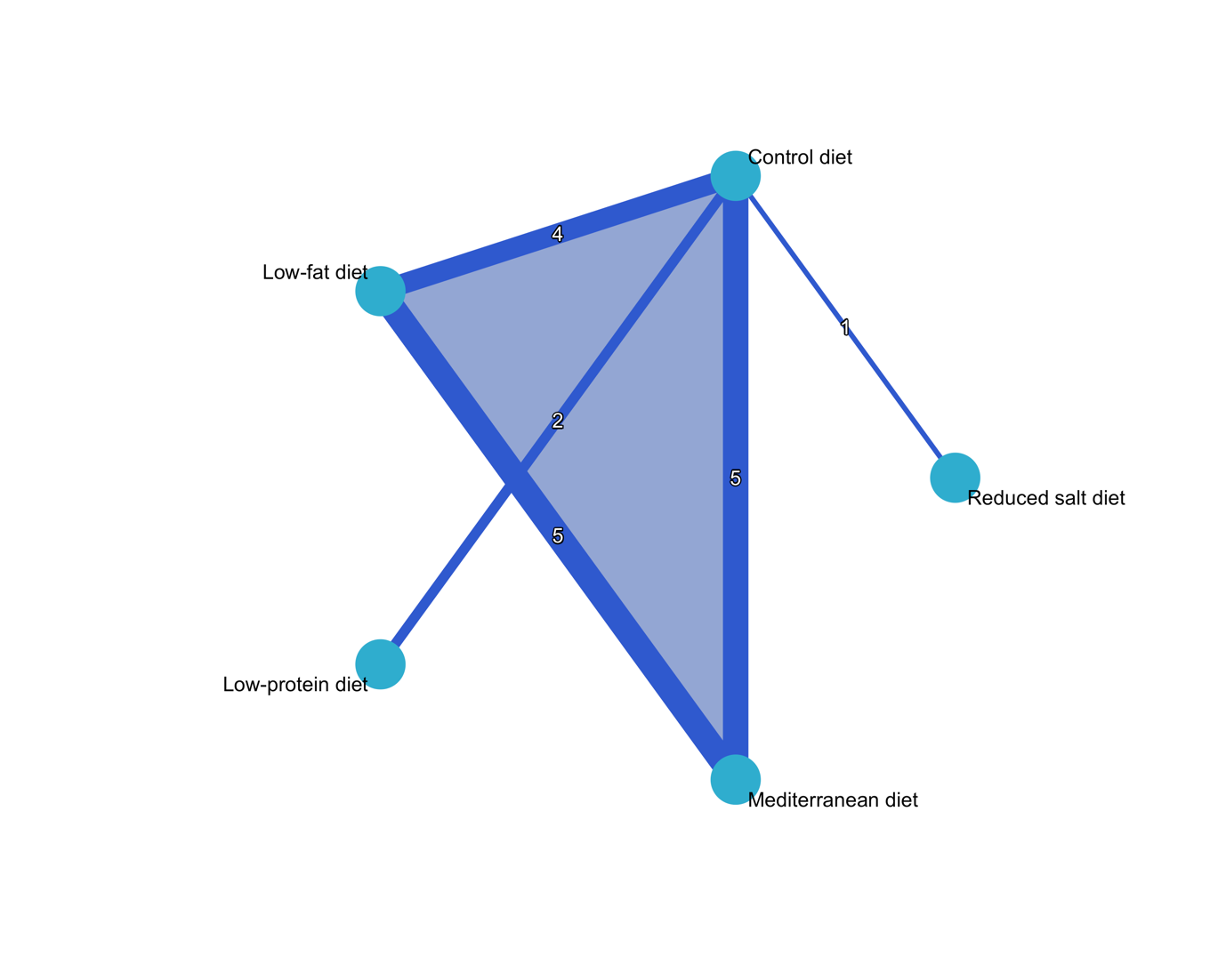


***Figure S5A.*** *Network graph of interventions for the mortality outcome.*

**Table S5A**. League table for the mortality

| Control Diet | 0.98 (0.74 – 1.30) | 0.89 (0.59 – 1.35) | **1.41 (1.06 – 1.87)** | 1.02 (0.64 – 1.62) |
| --- | --- | --- | --- | --- |
| 1.01 (0.80 – 1.26) | Low Fat Diet |  | **1.33 (1.03 – 1.70)** |  |
| 0.89 (0.59 – 1.35) | 0.89 (0.55 – 1.42) | Low Protein Diet |  |  |
| **1.37 (1.09 – 1.71)** | **1.36 (1.10 – 1.68)** | 1.53 (0.95 – 2.47) | Mediterranean Diet |  |
| 1.02 (0.64 – 1.62) | 1.01 (0.60 – 1.70) | 1.14 (0.61 – 2.14) | 0.74 (0.44 – 1.25) | Reduced Salt Diet |
| The upper triangle contains the pooled effect sizes of the direct comparisons available in our network. The lower triangle of the matrix contains the estimated effect sizes for each comparison. Significant results are in bold. | | | | |


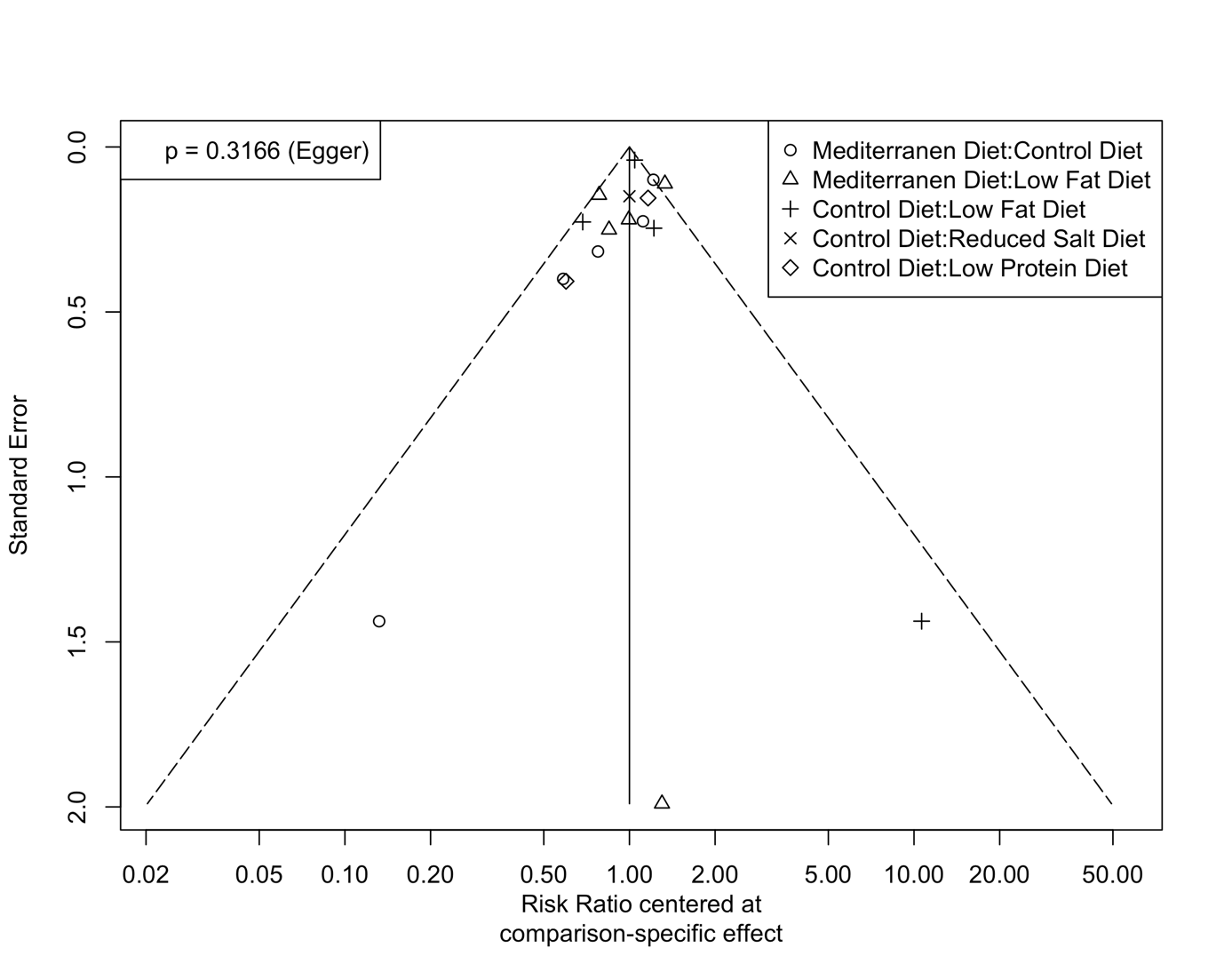


***Figure S5B.*** *Funnel plot of studies contributing to the network for the mortality outcome.*

**Supplementary material 6:** Network meta-analysis of interventions for the myocardial infarction


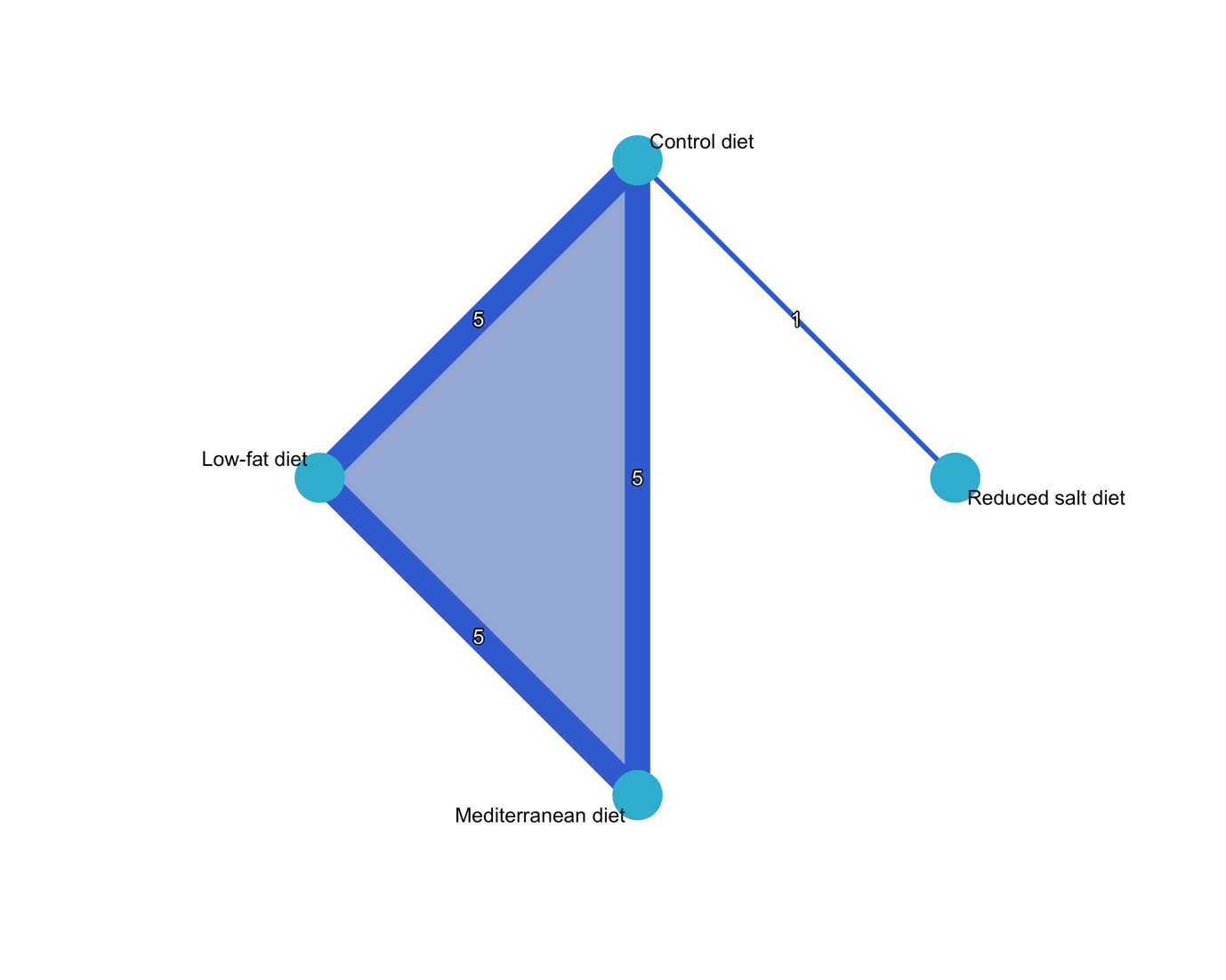


***Figure S6A.*** *Network graph of interventions for the myocardial infarction outcome.*

**Table S6A.** League table for the myocardial infarction

| Control Diet | 1.17 (0.87 – 1.56) | 1.36 (0.97 – 1.90) | 0.96 (0.48 – 1.94) |
| --- | --- | --- | --- |
| 1.09 (0.85 – 1.39) | Low Fat Diet | **1.43 (1.05 – 1.94)** |  |
| **1.48 (1.14 – 1.93)** | **1.36 (1.05 – 1.75)** | Mediterranean Diet |  |
| 0.96 (0.48 – 1.94) | 0.88 (0.42 – 1.86) | 0.65 (0.31 – 1.38) | Reduced Salt Diet |
| The upper triangle contains the pooled effect sizes of the direct comparisons available in our network. The lower triangle of the matrix contains the estimated effect sizes for each comparison. Significant results are in bold. | | | |

**
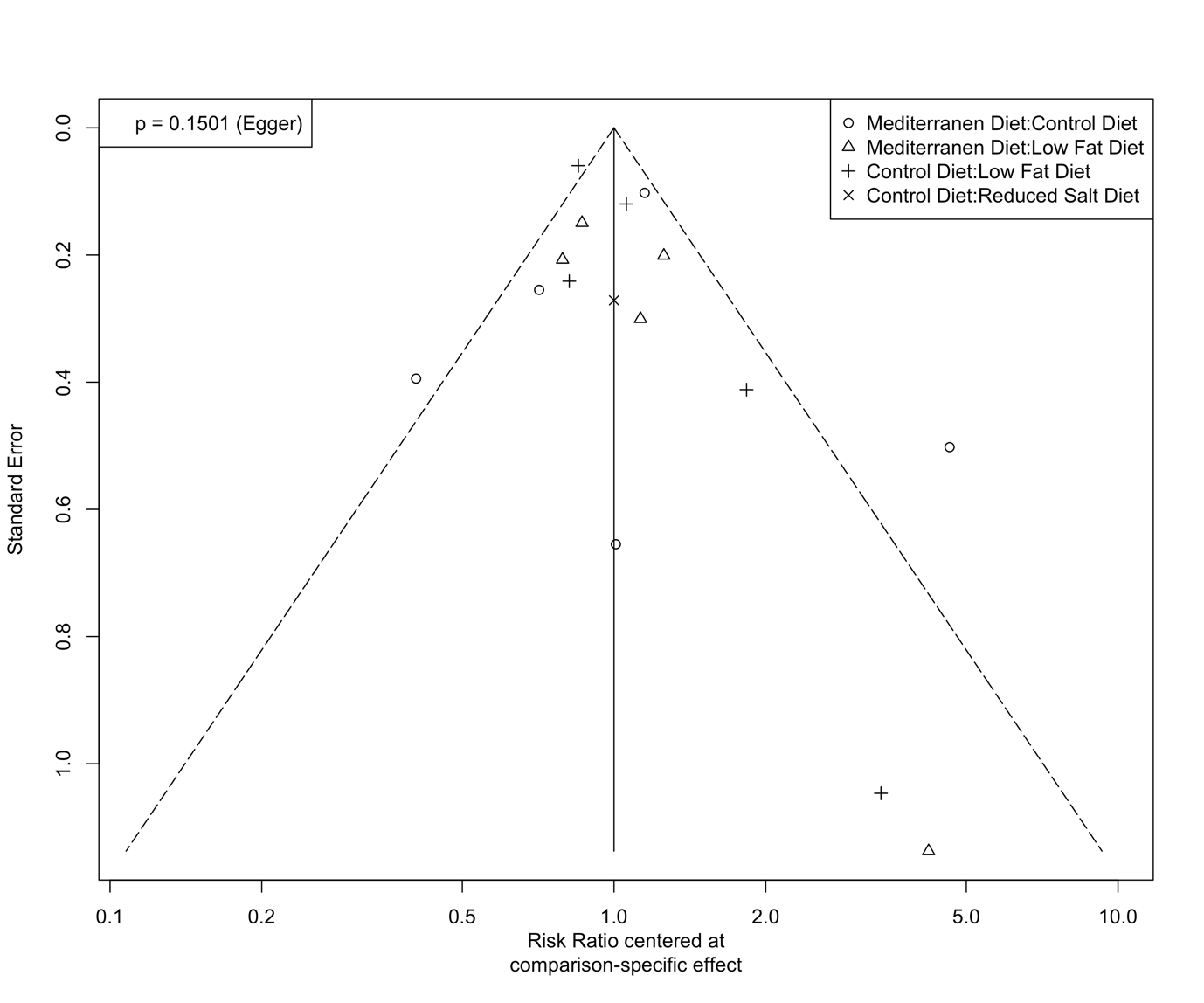
**

***Figure S6B.*** *Funnel plot of studies contributing to the network for the myocardial infarction outcome.*

**Supplementary material 7:** Network meta-analysis of interventions for the stroke


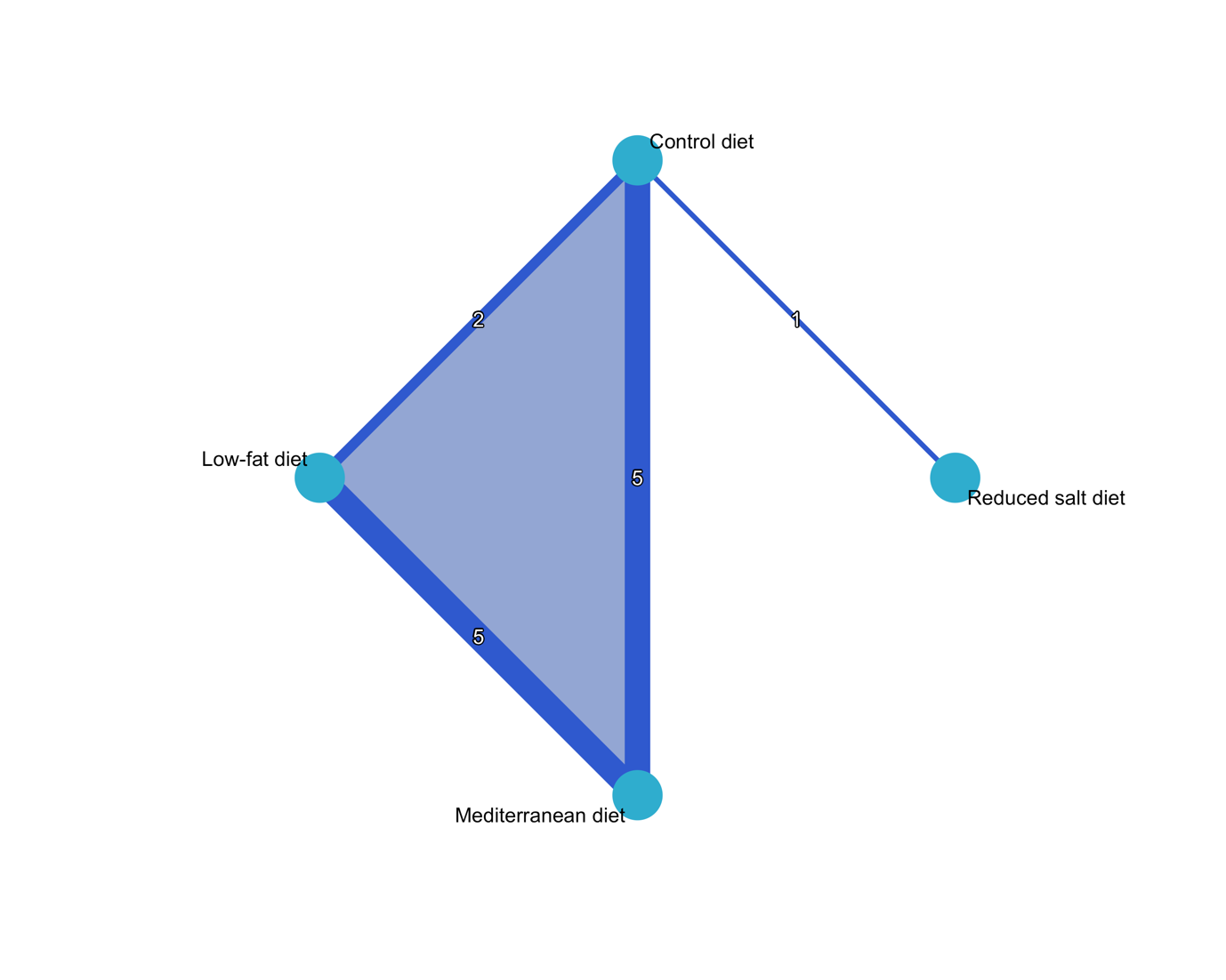


***Figure S7A.*** *Network graph of interventions for the stroke outcome.*

**Table S7A.** League table for the stroke

| Control Diet | 0.96 (0.78 – 1.18) | 1.02 (0.75 – 1.39) | 0.91 (0.67 – 1.24) |
| --- | --- | --- | --- |
| 0.89 (0.74 – 1.08) | Low Fat Diet | **1.57 (1.15 – 2.15)** |  |
| 1.19 (0.94 – 1.52) | **1.34 (1.05 – 1.70)** | Mediterranean Diet |  |
| 0.91 (0.67 – 1.24) | 1.02 (0.71 – 1.47) | 0.76 (0.52 – 1.13) | Reduced Salt Diet |
| The upper triangle contains the pooled effect sizes of the direct comparisons available in our network. The lower triangle of the matrix contains the estimated effect sizes for each comparison. Significant results are in bold. | | | |

**
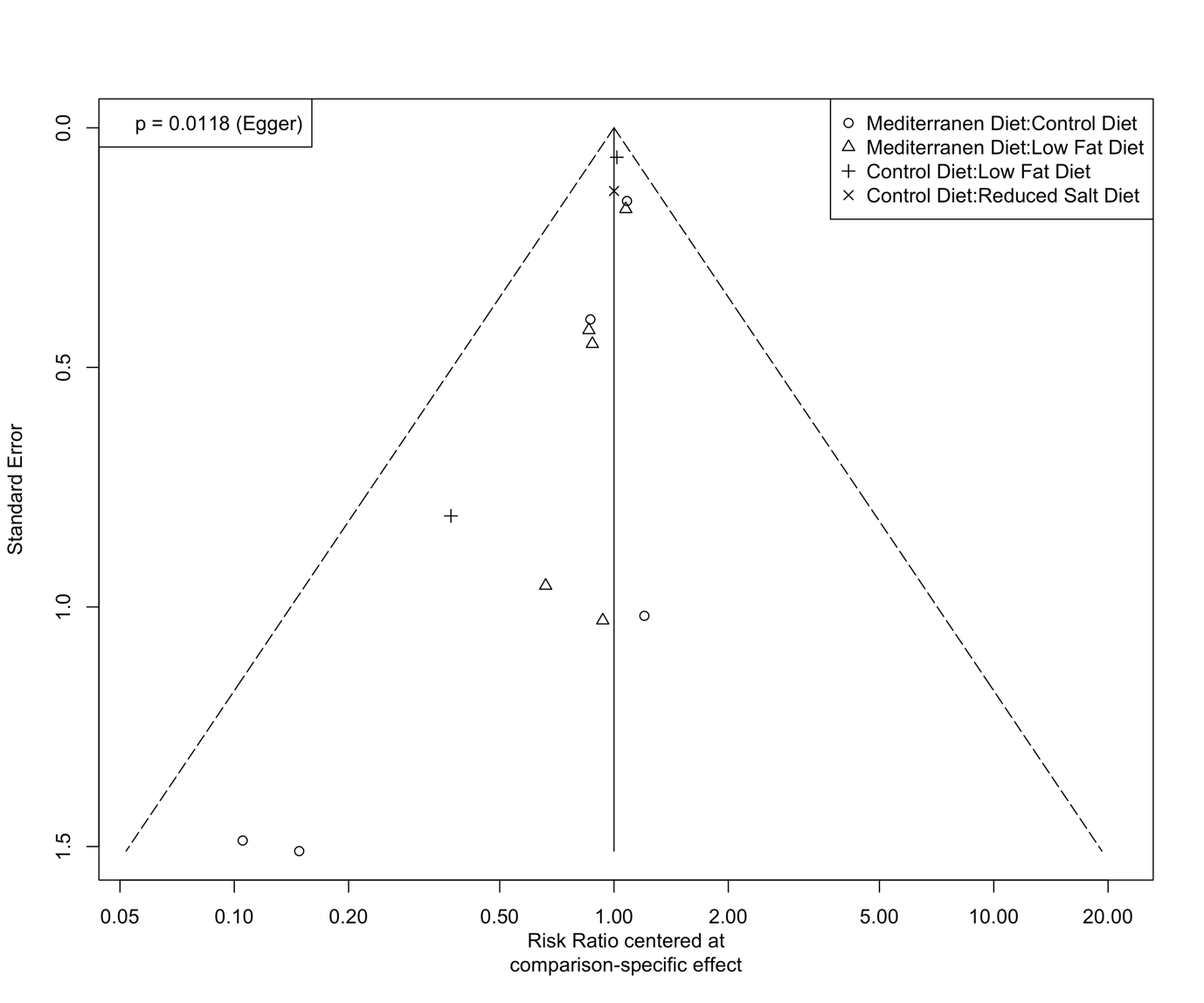
**

***Figure S7B.*** *Funnel plot of studies contributing to the network for the stroke outcome*

**Supplementary material 8:** Network meta-analysis of interventions for the major cardiovascular event (myocardial infarction, stroke, or death from cardiovascular causes) outcome


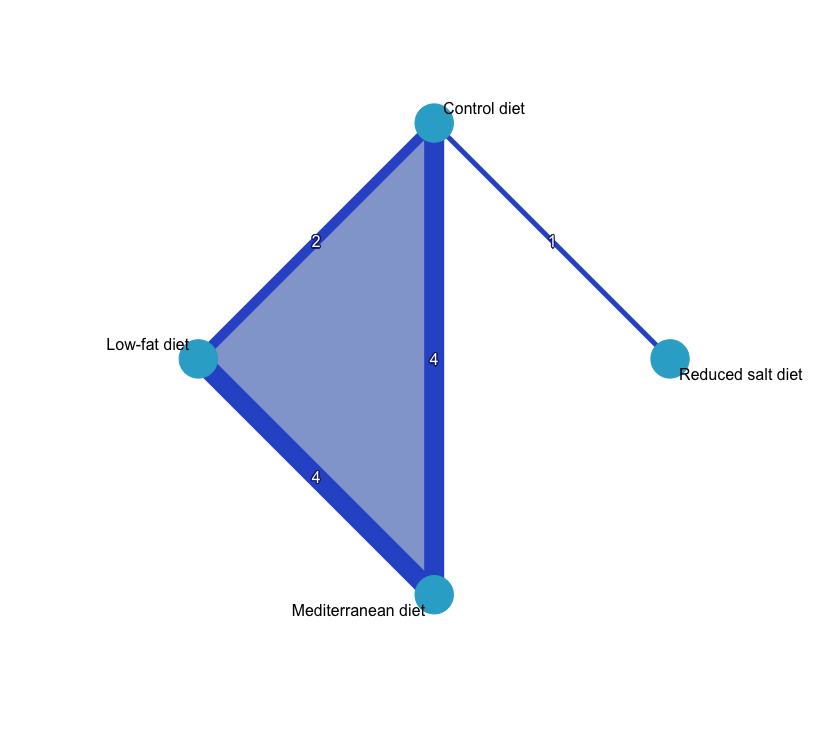


***Figure S8A.*** *Network graph of interventions for the major cardiovascular event outcome.*

**Table S8A.** League table for the major cardiovascular events

| Control Diet | 1.05 (0.69 – 1.61) | **1.55 (1.14 – 2.11)** | 0.96 (0.58 – 1.57) |
| --- | --- | --- | --- |
| 1.02 (0.76 – 1.38) | Low Fat Diet | **1.52 (1.14 – 2.03)** |  |
| **1.56 (1.19 – 2.04)** | **1.53 (1.18 – 1.97)** | Mediterranean Diet |  |
| 0.96 (0.58 – 1.57) | 0.94 (0.52 – 1.67) | 0.61 (0.35 – 1.07) | Reduced Salt Diet |
| The upper triangle contains the pooled effect sizes of the direct comparisons available in our network. The lower triangle of the matrix contains the estimated effect sizes for each comparison. Significant results are in bold. | | | |


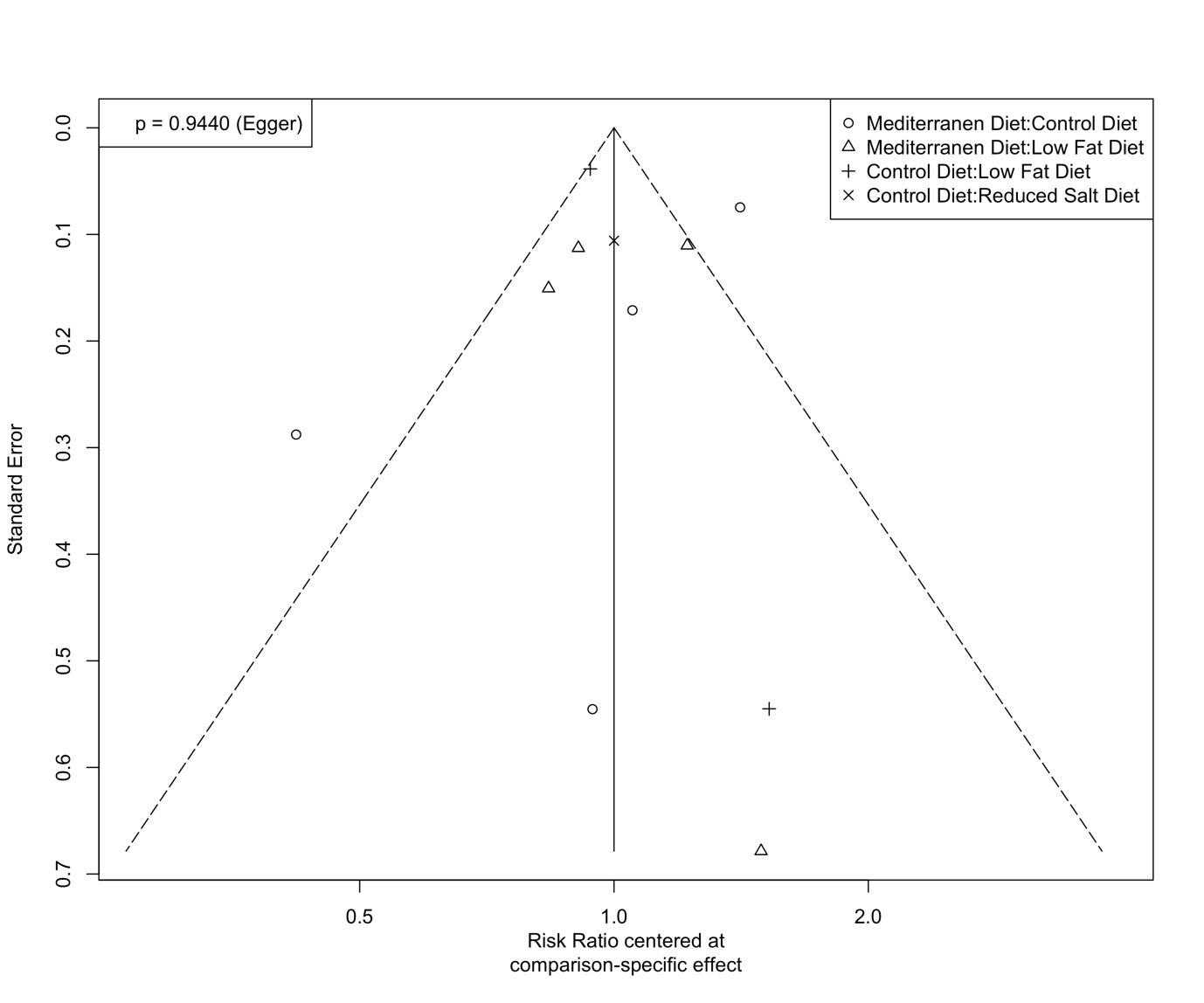


***Figure S8B.*** *Funnel plot of studies contributing to the network for the major cardiovascular event outcome.*

**Supplementary material 9:** Network meta-analysis of interventions for the angina outcome


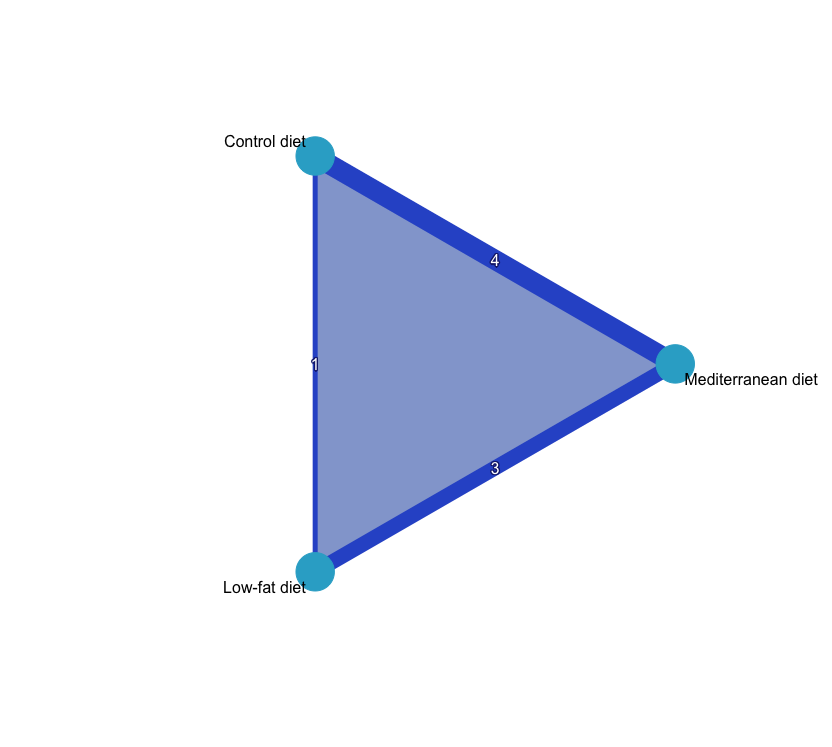


***Figure S9A.*** *Network graph of interventions for the angina outcome.*

**Table S9A.** League table for the angina

| Control Diet | 2.48 (0.41 – 14.98) | **2.50 (1.07 – 5.82)** |
| --- | --- | --- |
| 1.49 (0.48 – 4.63) | Low Fat Diet | 1.91 (0.73 – 5.03) |
| **2.72 (1.20 – 6.17)** | 1.83 (0.72 – 4.65) | Mediterranean Diet |
| The upper triangle contains the pooled effect sizes of the direct comparisons available in our network. The lower triangle of the matrix contains the estimated effect sizes for each comparison. Significant results are in bold. | | |

**Supplementary material 10:** Network meta-analysis of interventions for the heart failure outcome


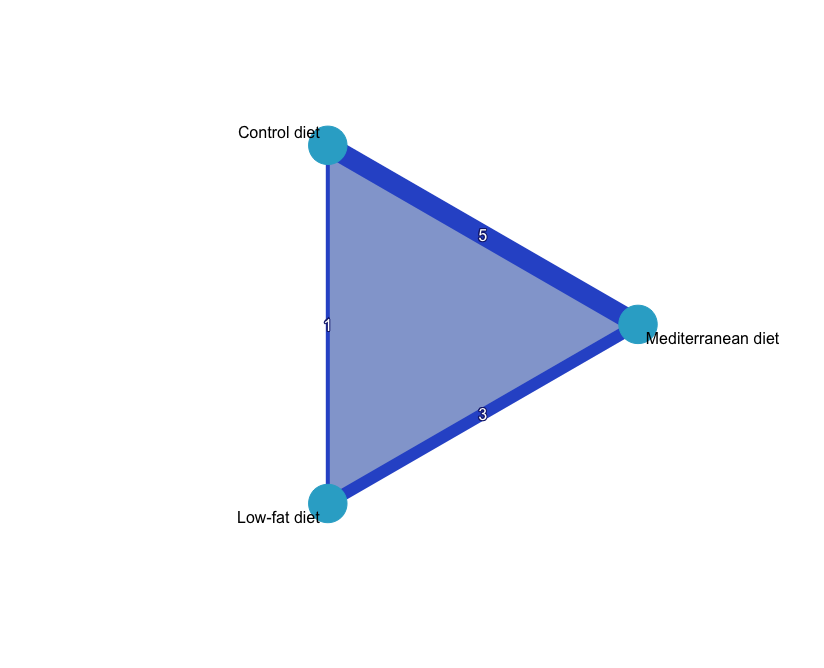


***Figure S10A.*** *Network graph of interventions for the heart failure outcome.*

**Table S10A.** League table for the heart failure

| Control Diet | 3.48 (0.17 – 70.88) | 1.50 (0.95 – 2.37) |
| --- | --- | --- |
| 0.98 (0.47 – 2.01) | Low Fat Diet | 1.63 (0.91 – 2.92) |
| 1.54 (0.98 – 2.41) | 1.58 (0.88 – 2.81) | Mediterranean Diet |
| The upper triangle contains the pooled effect sizes of the direct comparisons available in our network. The lower triangle of the matrix contains the estimated effect sizes for each comparison. Significant results are in bold. | | |

**Supplementary material 11:** Network meta-analysis of interventions for the atrial fibrillation outcome


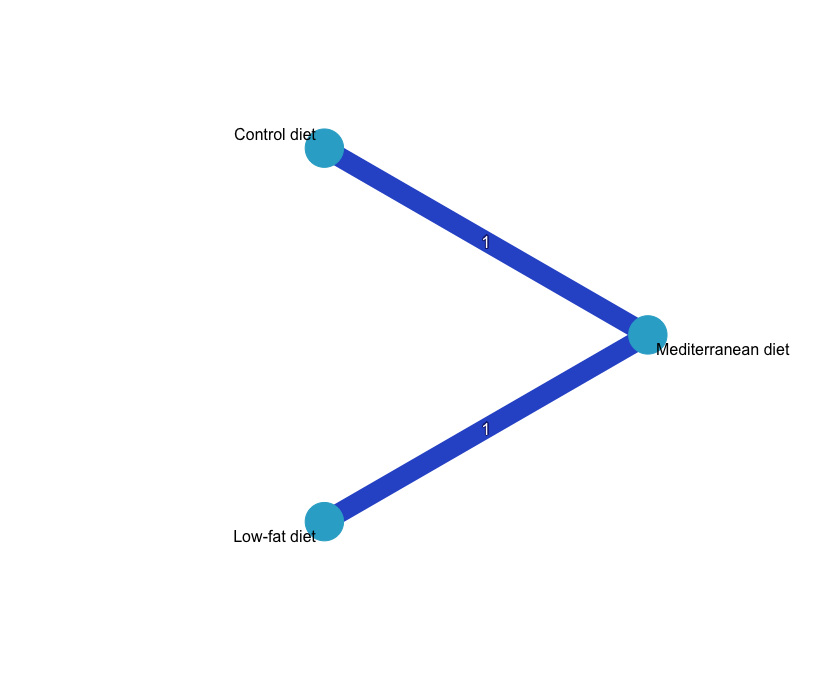


***Figure S11A.*** *Network graph of interventions for the atrial fibrillation outcome.*

**Supplementary material 12:** Grading of evidence for the primary outcome

| **Pairwise comparison** | **Network meta-analysis estimate** | **Confidence** | **Downgrading due to:** |
| --- | --- | --- | --- |
| **Mediterranean diet :**  **Control diet** | 0.57 (0.41 – 0.81) | Moderate | Heterogeneity |
| **Mediterranean diet :**  **Low fat diet** | 0.68 (0.49 – 0.94) | Low | Within-study bias  Imprecision  Heterogeneity |
| **Control diet : Low fat diet** | 1.18 (0.83 – 1.70) | Low | Within-study bias  Imprecision |
| **Control diet : Reduced salt diet** | 1.11 (0.55 – 2.24) | Low | Within-study bias  Imprecision |
| **Mediterranean diet : Reduced salt diet** | 0.64 (0.29 – 1.39) | Very low | Within-study bias  Imprecision |
| **Low fat diet : Reduced salt diet** | 0.94 (0.43 – 2.10) | Very low | Within-study bias  Imprecision |
